# Ectomycorrhizal Influence on Particle Size, Surface Structure, Mineral Crystallinity, Functional Groups, and Elemental Composition of Soil Colloids from Different Soil Origins

**DOI:** 10.1155/2013/698752

**Published:** 2013-05-23

**Authors:** Yanhong Li, Huimei Wang, Wenjie Wang, Lei Yang, Yuangang Zu

**Affiliations:** The Key Laboratory of Forest Plant Ecology Ministry of Education, Harbin, Heilongjiang 150040, China

## Abstract

Limited data are available on the ectomycorrhizae-induced changes in surface structure and composition of soil colloids, the most active portion in soil matrix, although such data may benefit the understanding of mycorrhizal-aided soil improvements. By using ectomycorrhizae (*Gomphidius viscidus*) and soil colloids from dark brown forest soil (a good loam) and saline-alkali soil (heavily degraded soil), we tried to approach the changes here. For the good loam either from the surface or deep soils, the fungus treatment induced physical absorption of covering materials on colloid surface with nonsignificant increases in soil particle size (*P* > 0.05). These increased the amount of variable functional groups (O–H stretching and bending, C–H stretching, C=O stretching, etc.) by 3–26% and the crystallinity of variable soil minerals (kaolinite, hydromica, and quartz) by 40–300%. However, the fungus treatment of saline-alkali soil obviously differed from the dark brown forest soil. There were 12–35% decreases in most functional groups, 15–55% decreases in crystallinity of most soil minerals but general increases in their grain size, and significant increases in soil particle size (*P* < 0.05). These different responses sharply decreased element ratios (C : O, C : N, and C : Si) in soil colloids from saline-alkali soil, moving them close to those of the good loam of dark brown forest soil.

## 1. Introduction

At the global scale, soil degradation, including soil erosion, is a potential threat to food security, and phytorehabilitation measures for controlling soil degradation are a popular and urgent topic of research [1, 2]. Ectomycorrhizal (ECM) fungi and associated symbionts can promote the growth of plants and increase their tolerance to unfavorable soil conditions such as nutrient deficiency or heavy metal pollution [3–5]. More than 5000 fungi can form ECM symbionts with over 2000 woody plants [6], showing the importance of ectomycorrhizae in plant-soil interactions. Commercially available mycorrhizal inocula, which consist of a single fungus species, are currently used for afforestation and grassland recovery [7]. ECM fungi enhance the growth and fitness of plants [8–11] by providing them with mineral and organic nutrients from the soil matrix and by protecting the carbohydrates and organic compounds that are stored in the roots from pathogenic organisms [12]. Improvements in root length, soil P utilization efficiency, and disease and stress resistance, as well as enhanced soil nutrient availability, have been reported [13, 14]. Recent studies have been performed on the identification of ECM fungi [15–18], interactions among various fungi and their effects on soil pollution rehabilitation [17, 19], and the underlying genetic basis for ECM functions [20–22]. These studies have provided a sound basis for understanding the mechanisms of the interaction between ECM fungi and various plants. Mycorrhizal fungi can also directly stabilize soil both through their hyphal network and through the secretion of glue-like chemicals [7]. However, the rarity of studies on the interaction between soil particles and ECM fungi hinders a full understanding of the function of fungi in soil health maintenance and soil physical texture formation. 

Owing to the profound heterogeneity among soil samples from field sampling campaigns, it is difficult to visualize the interaction between ECM fungi and soil aggregates at the microscale of millimeters or nanometers. Soil colloids are generally considered to be particles with effective diameters of around 10 nm to 10 *μ*m, with the smallest colloids just larger than dissolved macromolecules and the largest colloids being those that resist settling once suspended in soil pore water [23–25]. Soil colloids are the most active portion of the soil and largely determine the physical and chemical properties of the soil. Organic colloids are more reactive chemically and generally have a greater influence on soil properties per unit weight than inorganic colloids. Inorganic colloids of clay minerals are usually crystalline (although some are amorphous) and usually have a characteristic chemical and physical configuration. These features of soil colloids made it more suitable to study the interaction between ECM fungi and soil particles in indoor laboratory tests compared to bulk soils with heterogeneous composition [25]. In this paper, one aim was to find conformational changes in fungal extracts induced by the interaction with soil colloidal particles.

Previous papers have described good methods for identifying the surface structure and composition of soil colloids. To examine surface changes and microstructure changes in soil colloids after the addition of soil conditioner, Wang et al. used atomic force microscopy (AFM) to characterize 3D structural changes and used scanning electron microscopy (SEM) to find 2D surficial changes [26]. Laser particle-size analyzers have been used in a variety of studies with soil samples [27–30]. X-ray powder diffraction (XRD) is a nondestructive and rapid analytical technique primarily used for phase identification of soil minerals that can provide information regarding grain size and relative crystallinity, for example, interactions between clay minerals and organic matter in relation to carbon sequestration [31, 32]. Infrared spectroscopy is a well-established technique for the identification of chemical compounds and specific functional groups in compounds and, thus, is a useful tool for soil applications [33–35]. X-ray photoelectron spectroscopy (XPS) has the advantage of being able to detect all elements in the soil (except for H and He) and provides much valuable information on the composition of, and bonding state of elements in, surface and near-surface layers of many minerals [36–38]. The above methods may be useful for clarifying the influence of ECM fungus extracts on the surface structure and composition of soil colloids.

To reveal the underlying mechanism of the impact of ECM fungi on soil particles, soil colloids were extracted from 3 types of soils, including dark brown forest soil (deep and surface layers) and saline-alkaline soil in a grassland (surface layer). After fungal extract treatment, the laser particle-size analyzer, AFM, SEM, XRD, IR, and XPS were used to characterize the structural and surface changes. We hypothesized that ECM fungi would have different effects on soil colloids from different origins, and this could contribute to the improvement of degraded soil and the formation of healthy soil structure. The main aim of this study was to investigate the relationship between ECM fungi and soil and reveal the mechanism underlying soil improvement.

## 2. Material and Methods

### 2.1. Preparation of Soil Colloids

Soil samples were collected from the top soil (0–20 cm) in a typical saline-alkali region of the Songnen Plain (45°59′55′′N, 124°29′48′′E). As a nonsaline loam control, we used dark brown soil from the surface layer (0–20 cm) and a deeper layer (60–80 cm) from the Experimental Forest Farm of Northeast Forestry University (45°43′6′′N, 126°37′54′′E).

The soil colloids were separated according to [25, 26] as follows. One gram of air-dried soil was fully dispersed in 100 mL of ultrapure water in a 250 mL beaker. The suspension was allowed to stand undisturbed for more than 24 h. Sands and silts in the soil sample were gradually deposited at the bottom of the beaker, whereas the soil colloids were left in the suspension indicated as a turbid solution. The upper suspension was carefully decanted to a centrifuge tube and then centrifuged at 12000 rpm for 10 min. The precipitates were dissolved with 25 mL of ultrapure water and regarded as the soil colloidal solution. 

### 2.2. Preparation of Fungus Extract

*Gomphidius viscidus *was sampled from Inner Mongolia, China, for laboratory testing. It is an important mycorrhizal fungus in coniferous forests and belongs to the Gomphidiaceae family of the Agaricales [40]. It was grown in modified Melin-Norkrans (MMN) medium: CaCl_2_, 0.05 g; NaCl, 0.025 g; KH_2_PO_4_, 0.5 g; (NH_4_)_2_HPO_4_, 0.25 g; MgSO_4_ *·*7H_2_O, 0.15 g; FeCl_3_ (1%), 1.2 mL; thiamine HCl, 0.2 mL; malt extract, 3 g; glucose, 10 g; stock solution of micronutrients (contents per liter: H_3_BO_3_, 2.86 g; MnCl_2_, 1.81 g; ZnSO_4_, 0.22 g; CuSO_4_, 0.08 g; and NaMoO_4_, 0.02 g), 1 mL; ultrapure water, 1000 mL; and 15 g of agar in the case of agar media. The pH of the media was adjusted to 5.45–5.55 before autoclaving (121°C, 0.1 Mpa, and 20 min). The medium was dispersed aseptically in a 10 cm culture dish and stored in a 4°C refrigerator. The strain was inoculated on the MMN solid culture medium, training repeatedly until no other bacteria were produced [41].

Next, liquid medium was used, and 200 mL aliquots of the medium were dispensed into 250 mL beakers and autoclaved at 117°C for 20 min. Each Erlenmeyer flask was inoculated with fungal colonies of *G. viscidus* and then cultured on a shaking table. After 14 days, the fungal hyphae were used to extract fungal solution. First, culture media were filtered using a 4-layer gauze. After being cleaned repeatedly with ultrapure water, the mycelium was ground in a mortar and centrifuged (12000 rpm) at 4°C for 10 min, and the supernatant was collected as the fungus extract solution.

### 2.3. Particle Size and Surface Structure Observation

After the addition of fungus extract solution to soil colloids from different origins, the particle size and surface structure were determined with a laser particle-size analyzer (ZetaPALS, USA), an atomic force microscope (AZ, USA), and a scanning electron microscope (Quanta 200, FEI, USA). Methods were revised from [26, 30], and detail was as follows.

For the laser size analysis, 10 *μ*L of fungus extract was added to 4 mL of soil colloid solution (about 1 mg soil colloids), and the fungus extract was replaced by 10 *μ*L of ultrapure water in the control group. Twelve hours after mixing, the mixture was analyzed using the laser particle-size analyzer. For AFM observation, 10 *μ*L of fungus extract was added to 4 mL of soil colloid solution, and the fungus extract was replaced by 10 *μ*L of ultrapure water in the control group. One hour after mixing, centrifugation (12000 rpm) at 25°C for 10 min was used to precipitate the soil colloids. This colloid precipitate was then dissolved with 4 mL of ultrapure water, dropped on the mica surface, and air-dried before AFM imaging. The AFM images of the soil colloids were obtained in tapping mode by using the PicoPlus II AFM system from Molecular Imaging (MI) Corporation (AZ, USA). For SEM observation, the same procedures as those for AFM observation were used, and the soil colloidal solution was dropped on the sample stage and then air-dried before SEM imaging (Quanta 200, FEI, USA). The samples were sputter-coated with a thin layer of gold-palladium (5–10 nm, 25 mA, and 3 min) at room temperature by using a sputter coater before the examination. 

### 2.4. Crystal Structure Diffraction of Soil Minerals

The preparation of treatments and controls was the same as that for AFM, and SEM and X-ray powder diffraction (XPD) observation were carried out according to [32]. After being air-dried, X-ray powder diffraction patterns were collected in transmission by using an X-ray powder diffractometer (D/Max 2200, Rigaku, Japan) with a rotating anode (Philips) and Cu K*α*_1_ radiation generated at 30 mA and 40 kV. The range of 2*θ* diffraction angles examined was 10–40° with steps of 0.02° and a measuring time of 0.3 s per step.

### 2.5. Observations of Functional Group Change

The preparation of treatments and controls was the same as that for AFM, and SEM and infrared spectrum (IR) measurements were carried out according to [33–35]. The samples were diluted with 1% KBr mixing powder and separately pressed to obtain self-supporting disks. Tablets for IR measurements were prepared by pressing the powder mixture at a load of 8 tons for 8 min. The IR spectrum was obtained by a compact Fourier transform infrared spectrophotometer (IR Affinity-1, SHIMADZU, Japan) and recorded across a wave number range of 4000–500 cm^−1^ at a resolution of 4 cm^−1^.

### 2.6. Analysis of the Atomic Concentration of Elements

A K-Alpha spectrometer equipped with a concentric hemispherical analyzer in the standard configuration (Thermo Scientific, USA) was used in this analysis with the method revised from [38]. After the preparation of control and treatment samples (the same as that for SEM and AFM), the soil colloidal solution was dropped on a cleaned HOPG surface and then air-dried before examination. The vacuum system consisted of a turbomolecular pump and a titanium sublimation pump. The residual pressure before the analysis was lower than 10^−7 ^Pa. The X-ray source was AlK*α*, and it was run at 30 mA and 80 kV. The incident angle was 49.1°, and the emission angle was 0° with respect to the sample's surface normal. All the spectra were obtained in digital mode. The wide-scan spectra were acquired from 1000 eV to 0 eV. Sample charging was corrected by comparing all binding energies to the adventitious carbon at 285.0 eV. Detailed spectra were processed using CasaXPS software (V2.3.12, Casa Software Ltd., UK). An iterated Shirley-Sherwood background subtraction was applied before peak fitting using a nonlinear least-squares algorithm. The atomic concentration of elements was calculated using the software.

### 2.7. Data Analysis

In the analysis of XRD data, the original data were rectified using the Jade program to eliminate K*α* and then obtain the XRD pattern for a sample. The upper area (*a*_*c*_), which was separated with the smooth curve connecting each point of minimum intensity, corresponded to the crystalline portion, and the lower area was the background containing the amorphous portion (*a*_*b*_). The Jade program was used to calculate grain size and relative crystallinity (relative crystallinity = *a*_*c*_/(*a*_*c*_ + *a*_*b*_) [42] and check the effect of the addition of fungus extracts. The major components of the soil were obtained in conjunction with the database and according to the books written by Xie [43] and Marc and Jacques [44].

Six functional groups [45] were selected for IR analysis, as shown in Figure 5: 3750–3300 cm^−1^ is O–H stretching of structural OH; 1200–970 cm^−1^ is Si–O–Si stretching; 950–820 cm^−1^ is O–H bending of structural OH; 2970–2820 cm^−1^ is aliphatic C–H stretching; 1750–1630 cm^−1^ is C=O stretching of carboxylic acids, amides, and ketones; and 1650–1360 cm^−1^ is carbonates.

## 3. Results

### 3.1. Results Obtained Using the Laser Particle-Size Analyzer

**Figure 1:**
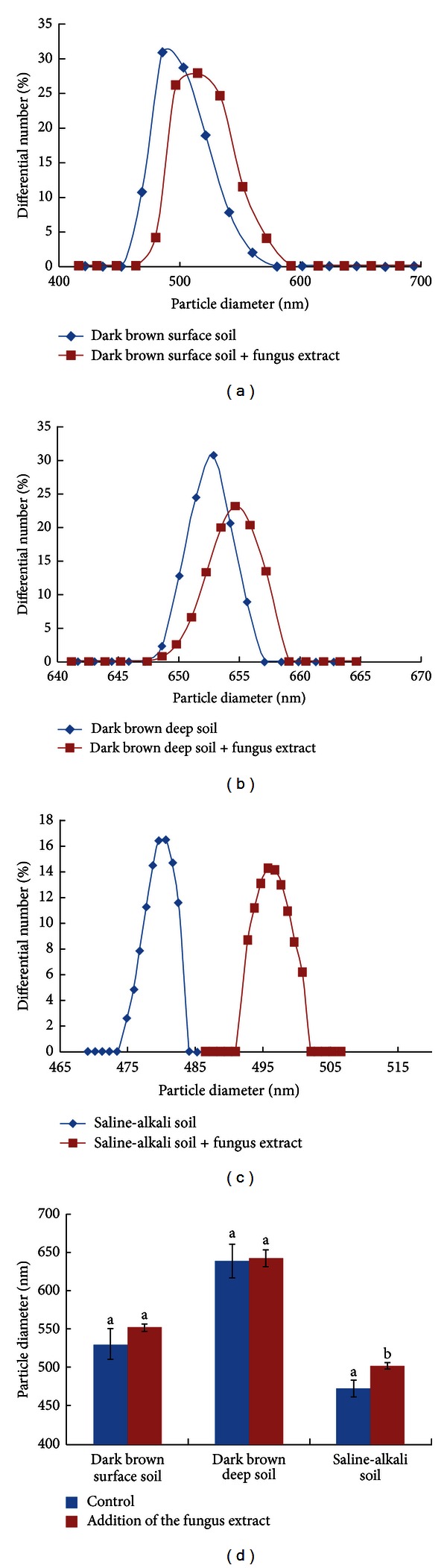
Particle diameter changes in soil colloids with and without fungus extract addition.

The particle size and distribution of soil colloids from dark brown forest soil (surface and deep layers) and saline-alkali soil with and without the addition of fungus extracts are shown in Figure 1. After the addition of the fungus extract, the particle size of soil colloids from dark brown forest soil in the surface layer was larger than that in the control, and similar results were found for deep soils. However, the differences were not significant between the treatment and the control (*P* > 0.05). In contrast, the addition of the fungus extract increased the particle size of colloids from saline-alkaline soil from 472 ± 11.3 nm to 502 ± 4.0 nm, and this 6.29% increase was statistically significant (*P* < 0.05) (Figure 1).

### 3.2. AFM Results

**Figure 2:**
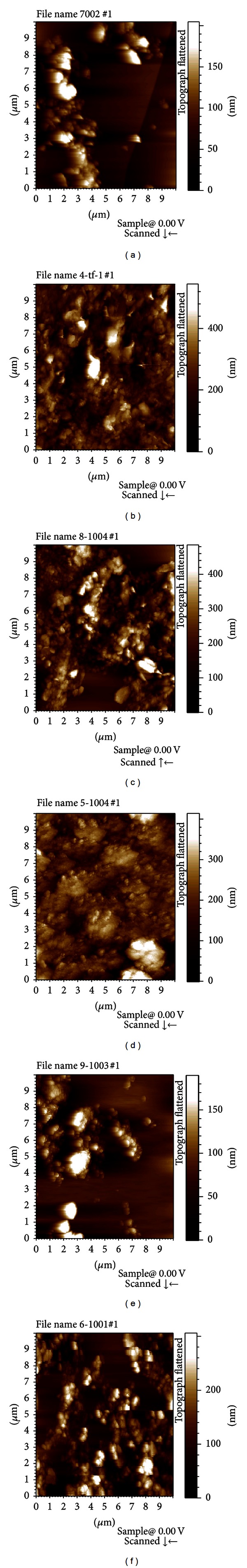
Atomic force microscopy images of soil colloids with (b, d, and f) and without (a, c, and e) fungus extract addition. (a) Colloids from the surface layer of dark brown forest soil; (b) colloids from the surface layer of dark brown forest soil + fungus extract; (c) colloids from the deep layer of dark brown forest soil; (d) colloids from the deep layer of dark brown forest soil + fungus extract; (e) colloids from saline-alkali soil; (f) colloids from saline-alkali soil + fungus extract.

As shown in Figure 2, soil colloids from the surface and deep layers of dark brown forest soil showed similar tendencies. More soil colloids aggregated together and became larger than those in the control. Moreover, more covering materials were found on the surface of these colloids than those in the control samples. Contrary to the soil colloids from the dark brown forest soil, soil colloids from the saline-alkali soil were dispersed after the addition of fungus extracts, and the interaction between the different particles was not as dense as that in the control (Figure 2). 

### 3.3. SEM Results

**Figure 3:**
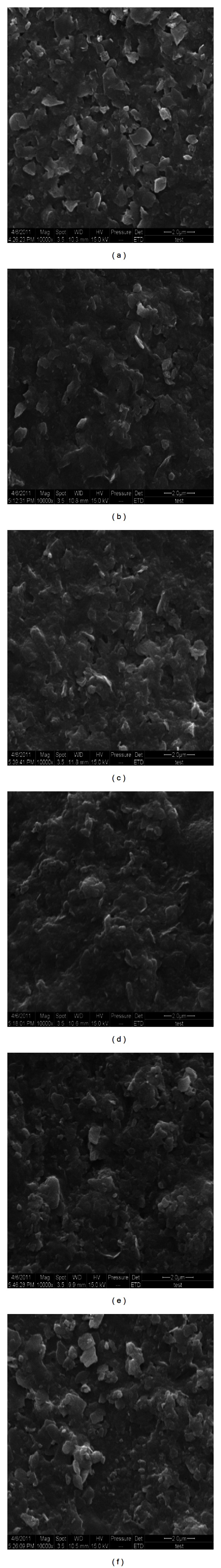
Scanning electron microscopy images of soil colloids with (b, d, and f) and without (a, c, and e) fungus extract addition. The labels are the same as those for Figure 2.

The results of SEM were similar to those of AFM (Figure 3). After the addition of the fungus extract, the edges of colloid particles from the dark brown forest soil became much smoother, the small gap between soil colloid particles became invisible, and some covering materials seemed to fill these gaps and made the edges less distinct. This tendency was more evident in surface soils than in deep soils (Figures 3(a)–3(d)).

However, results from the saline-alkali soil were different from those from the dark brown forest soil; relatively larger particles with more distinct and acute edges were observed after the addition of the fungus extract (Figures 3(e) and 3(f)).

### 3.4. XRD Results

**Figure 4:**
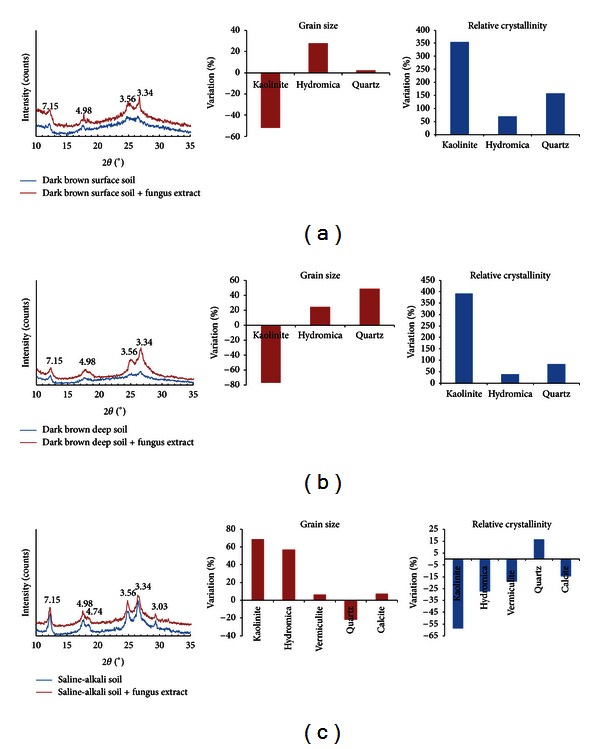
X-ray powder diffraction results with and without fungus extract addition. (a) Colloids from the surface layer of dark brown forest soil; (b) colloids from the deep layer of dark brown forest soil; (c) colloids from saline-alkali soil.

Both the surface and deep layers of dark brown forest soil had 4 obvious diffraction peaks located at 12.3°, 17.8°, 25.0°, and 26.7°. These peaks are indicative of 3 kinds of minerals: kaolinite (7.15 Å, 3.56 Å), hydromica (4.98 Å), and quartz (3.34 Å) (Figures 4(a) and 4(b)). Soil colloids from saline-alkali soil had 6 diffraction peaks, located at 12.3°, 17.8°, 18.6°, 25.0°, 26.7°, and 29.3° indicating that the main soil mineral composition was kaolinite (7.15 Å, 3.56 Å), hydromica (4.98 Å), vermiculite (4.74 Å), quartz (3.34 Å), and calcite (3.03 Å) [44] (Figure 4(c)).

The addition of the fungus extract increased the relative crystallinity of soil colloids from both the surface and deep layers of dark brown forest soil (Figures 4(a) and 4(b)). Kaolinite increased by 300%, hydromica by 40–70%, and quartz by 83–157%. The grain size of hydromica and quartz both increased by 3–25%, but the kaolinite was reduced by 50–77%.

Unlike with dark brown forest soil, the addition of fungus extracts decreased the relative crystallinity of soil colloids from saline-alkali soil, except for quartz (a 17% increase) (Figure 4(c)). The largest reduction was 59% in kaolinite, while the decreases for hydromica, vermiculite, and calcite ranged from 15% to 25%. In most cases, the grain size of these soil minerals increased after the addition of the fungus extract, except for quartz (reduced 22%) (Figure 4(c)). Grain size increased by about 7% in vermiculite and calcite, while much larger increases (over 57%) were found in hydromica and kaolinite (Figure 4(c)).

### 3.5. IR Results

**Figure 5:**
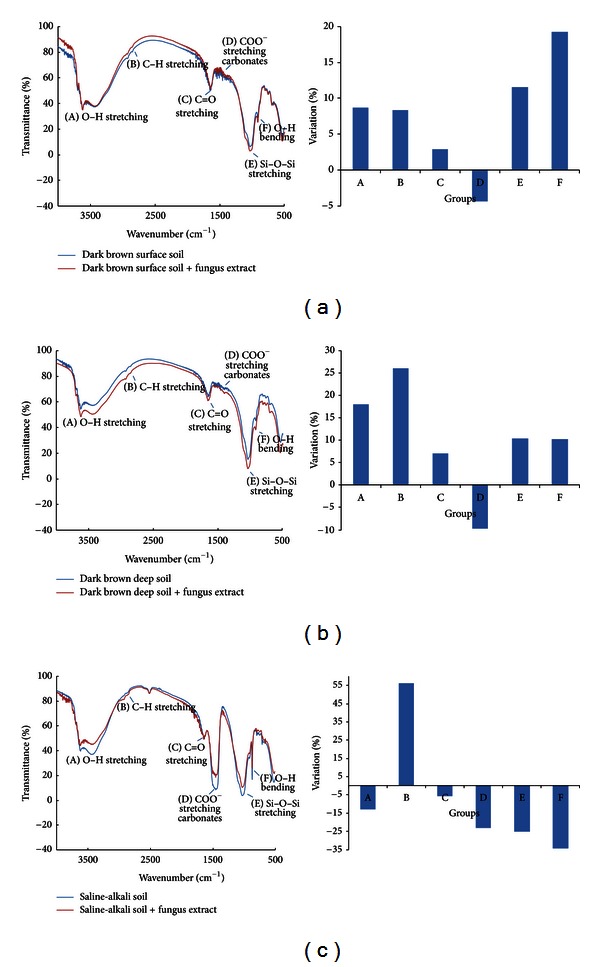
Infrared spectrum results after fungus extract addition. The labels are the same as those for Figure 4.

The addition of the fungus extract to soil colloids from the dark brown forest soil slightly reduced the amount of stretching of the COO^−^ and carbonate functional groups (about 10%) but increased O–H bending, increased stretching in most of the studied functional groups, including C–H, Si–O–Si, and O–H (all about 10–20%), and slightly increased (5%) C=O stretching (Figure 5(a)). Similar tendencies but slight differences in the size of changes were found in deep soil than in surface soil (Figure 5(b)). 

Most functional group traits in the surface and deep layers of dark brown forest soil increased. However, a completely different pattern was found in saline-alkali soil (Figure 5(c)). In contrast with dark brown forest soil, the addition of fungus extracts to soil colloids from saline-alkali soil reduced the traits of most functional groups from 10% to 35% (Figure 5(c)). Functional group traits that decreased included O–H bending, C=O stretching, Si–O–Si stretching, O–H stretching, COO^−^ stretching, and carbonate stretching, with the exception of C–H stretching (a 56% increase) (Figure 5(c)). 

### 3.6. XPS Results

**Figure 6:**
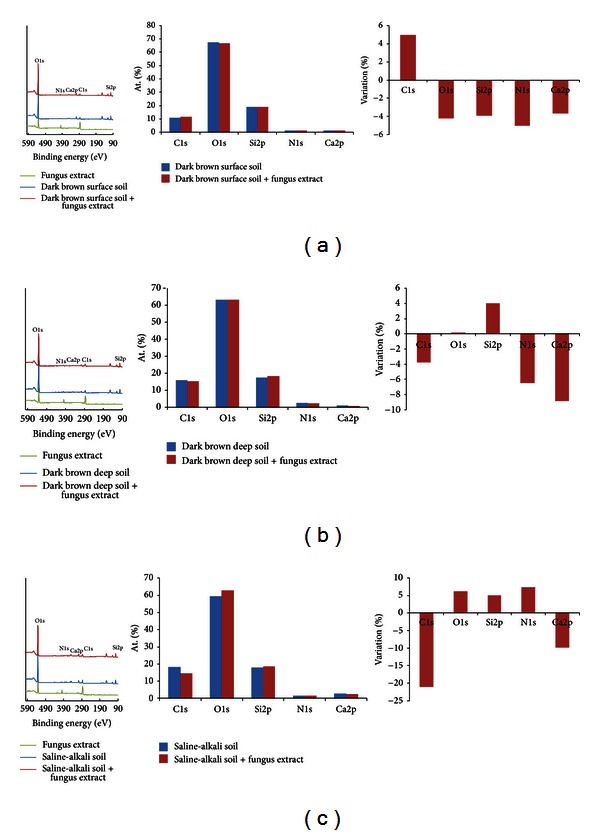
X-ray photoelectron spectroscopy results with and without fungus extract addition. The labels are the same as those for Figure 4.

Semiquantitative analysis of variable elements with and without the addition of the fungus extract was performed using XPS (Figure 6). In the case of soil colloids from the surface layer of dark brown forest soil, the addition of the fungus extract induced <5% changes in all elements, for example, a 5% increase in C1s and <5% decreases for all O1s, Si2p, N1s, and Ca2p (Figure 6(a)). Changes in variable elements in the deep soil due to the addition of fungus extracts were more evident than those in the surface layers (Figures 6(a) and 6(b)). The changes in C1s, O1s, and Si2p were less than 5%, while 6–9% decreases in N1s and Ca2p were observed (Figure 6(b)). 

Compared to the dark brown forest soil, addition of the fungus extract to the saline-alkali soil caused large reductions in variable elements (Figure 6(c)). C1s decreased by 21%, Ca2p by 10%, and O1s, Si2p, and N1s by 5%.

Stoichiometric changes induced by fungus extract addition were also found in the ratios among different elements (Table 1). In the case of the surface layer of dark brown forest soil, the ratios of C : N, Si : Ca, and C : Ca increased by 7–14%. In the case of the deep layer of dark brown forest soil, changes were also mainly found in C : N (6.5%), Si : Ca (20.5%), and C : Ca (17.1%). Stoichiometric changes were much more evident in saline-alkali soil than in dark brown forest soil. Over 25% decreases were found in C : O, C : N, and C : Si, and a 12.7% decrease was found in C : Ca. The Si : Ca ratio increased by 16.39% (Table 1).

## 4. Discussion

**Figure 7:**
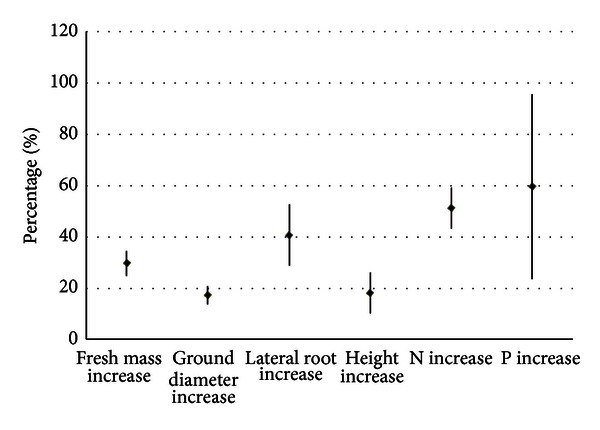
ECM infection influences on plant growth and biomass N, P absorption [39]. The ECM is the same species, *Gomphidius viscidus*, used in this study, and plant species are *Larix kaempferi*, *Pinus tabulaeformis*, and *Quercus liaotungensis*. The percentage in *y*-axis is the improvement of each parameter relative to the untreated control (without ECM infection).

Heavy soil degradation is common in China, and rehabilitation via vegetation recovery is mainly conducted in degraded regions, such as the saline-alkali soil region in the Songnen Plain [26]. Symbiotic associations between tree roots and ECM fungi play important roles in promoting the growth of these plants, and this promotion can be affected by variation in the strains of ECM fungi [46] and their interactions between biotic and abiotic factors [47]. ECM can aid the recovery of degraded soil both through the direct absorption of variable pollutants [17, 19] and indirect protection from rehabilitated vegetation [41]. In a previous study of ECM fungi, the main focus was on the relationship between ECM and plants. In the case of the ECM studied in this paper (*Gomphidius viscidus*), reviewed data have found significant increase in biomass of variable trees together with more soil nutrient absorption (Figure 7): that is, growth of biomass, ground diameter, lateral root and height of larch, pine, and oak increased from 20% to 40%, and much higher increase (50%–60%) in N, P absorption was found. This result indicates that soil nutrient absorption increase should be a basis for the biomass increase after ECM infection. However, few studies paid attention to the underlying mechanism of the interaction between ECM and the soil matrix. The findings in this paper showed that the ECM influences on soil colloids should be important aspect of degraded soil improvement.

Dark brown forest soil and saline-alkali soil are two types of soil that are widespread in northeastern China. The first is an example of loam with good physicochemical properties [48], while the latter is notorious for poor physicochemical properties because of long-term degradation from human disturbance [26]. In the case of dark brown forest soil from surface and deep layers, both AFM and SEM images revealed that viscous materials wrapped around soil particles, filled some gaps among particles, and induced smoother surfaces with unclear edges (Figures 2(a)–2(d) and Figures 3(a)–3(d)). High absorption capacity is the basis for adhesive material absorption on soil colloids. This absorption has been reported previously [23]. Yan et al. reported that the maximum adsorption capacity (*q*_0_) for fine soil colloids ranged from 169.6 to 203.7 *μ*g mg^−1^ [49], which was higher than that for coarse soil colloids (81.0–94.6 *μ*g mg^−1^). Thus, physical absorption instead of chemical reactions possibly occurred in dark brown forest soil. 

However, saline-alkali soil was different from dark brown forest soil. There were no clear adhesive layers on soil colloids, and smoother edges were found after the addition of fungus extracts (Figures 3(e) and 3(f)). Significantly larger particle sizes (Figure 1) and relatively loose interactions between different soil particles were observed in saline-alkali soil colloids (Figures 2(e) and 2(f)). Saline-alkali soil has a clay texture with tiny soil particles, high pH, and rich carbonates (especially calcium carbonate) [26]. This soil has poor soil physical structure with limited soil aggregates, and soil colloid surfaces are overloaded by Na^+^ [48]. Wang et al. (2011) reported that the addition of the soil conditioner HPMA induced flocculation of soil colloids with looser structure and larger aggregates [26]. The findings on AFM, SEM images, and soil particle size indicate that the function of the fungus extract is like that of the soil conditioner (e.g., HPMA [26]), and a chemical reaction instead of physical adhesion may occur between the fungus extract and soil colloids. The mechanism of saline-alkali soil improvement by HPMA conditioner is to activate inorganic calcium in the soil via a chemical reaction between calcium carbonate and HPMA, as well as the exchange of Ca^2+^ and excessive Na^+^ on the surface of soil colloids [26]. A similar chemical reaction is possibly activated after the addition of the fungus extract to soil colloids from saline-alkali soils, owing to the fact that organic acids were present in the fungus extract solution. 

It is worth mentioning that carbonate functional groups appear obviously reduced by 25%, perhaps because saline-alkali soil is basically an alkaline soil with a pH of 9-10 and includes more carbonate (CaCO_3_) in calcite, while the fungus extract is generally acidic; therefore, combining them may cause some chemical reaction and lead to calcium carbonate being dispersed. Dissolution of calcium carbonate could lead to more Ca^2+^ being adsorbed on the surface of soil colloids, which would tend to increase soil colloid dispersion and form a more loose soil colloid apparent structure [26], as shown in Figure 2.

Measuring differences in mineral composition on the surface of soil colloids and in mineral crystallinity may help explain the changes in particle diameter and surface structure as shown in Figures 1–3. The techniques of XRD, IR, and XPS are commonly used for mineral crystallinity observation [42, 43], functional group identification in soils [44], and elemental composition changes [50]. These methods could be used to examine mineral crystallinity changes, variable functional group changes, and the elemental composition of soil colloids, together with surface and particle size changes after the addition of the fungus extract.

XRD data proved that fungus extract treatment could change the relative crystallinity and grain size of variable soil minerals and showed differences between dark brown forest soil and saline-alkali soil (Figure 4). In the case of dark brown forest soil, addition of fungus extracts 30%~400% increased crystallinity of kaolinite, hydromica, and quartz, while 17%~58% decreases were found in saline-alkali soil (except for a 15% increase for quartz) (Figure 4). These data were well-matched with the observations from SEM and AFM; that is, soil colloid particle edges became blurred with covering materials in dark brown forest soil, while soil colloids from saline-alkali soil tended to be dispersed with relatively loose interactions (Figures 2–3). By using a similar XRD method, Zheng and Zhao (2011) found that soil clay minerals (illite, kaolinite, and montmorillonite) are very sensitive to disturbance, and their changes could affect soil fertility [51]. Our results showed that ECM fungi in soil may favor this process via their effect on the relative crystallinity and grain size of soil minerals.

IR can provide detailed information about variable functional groups [44], and IR data from this study showed ECM fungus could induce evidently different responses between saline-alkali soil and dark brown forest soil. Traits of functional groups such as O–H stretching and bending, C–H stretching, and Si–O–Si stretching in soil colloids of dark brown forest soil were increased by 8–27% after the addition of the fungus extract (Figures 5(a) and 5(b)). However, all the functional group traits in saline-alkali soil were reduced by about 10–35%, except for C–H stretching. The changes in functional group composition (Figure 4) and mineral crystallization (Figure 5) in soil colloids were accompanied by relative changes in element composition (Figure 6 and Table 1), too. Compared with very slight changes in dark brown forest soil, addition of the fungus extract to degraded soil (saline-alkali soil) majorly reduced C1s (21%) and made the C : O, C : N, C : Si, and C : Ca element ratios more similar to those of high-quality dark brown forest soil (Table 1). ECM inoculum has been commonly used during forestry plantings and native prairie restorations to enhance tree and plant growth [11]. 

The present study clarifies the mycorrhizal-soil interaction. Field studies indicate that mycorrhizal inocula benefit native plant production and establishment in severely degraded areas [11, 52], and commercial mycorrhizal-soil conditioners are now available for vegetation recovery [7]. The interactions between ECM fungus extracts and soil colloids observed in this study indicate that there are relatively fast physical and chemical reactions between fungi and tiny soil particles and that the clarification of this process may promote the understanding of the function of ECM in soil nutrient exploitation and soil health maintenance. 

## 5. Conclusions

The addition of fungus extract to soil colloids could significantly affect the surface structure, mineral crystallization, functional groups, and elemental composition of colloids, although the effects were different for dark brown forest soil (a good loam) and saline-alkali soil (a heavily degraded soil). For dark brown forest soil, physical absorption at the surface of soil colloids together with increases in functional group traits such as O–H stretching and bending, C–H stretching, C=O stretching, and Si–O–Si stretching and the relative crystallinity of kaolinite, hydromica, and quartz were observed after the addition of fungus extracts. In the case of degraded saline-alkali soil, the particle diameter and grain size of variable soil minerals were sharply increased, and remarkable reductions were found in the relative crystallization of variable minerals and most functional groups (5 out of 6), showing that chemical reactions instead of physical absorption possibly occurred after the addition of the fungus extract. As good loams and degraded soils are typical in northeastern China, the findings in this paper will improve the understanding of the mechanisms for ECM-aided soil improvements, especially for highly degraded soil.

## Figures and Tables

**Table 1:** Results from X-ray photoelectron spectroscopy of the variation in element ratios with and without the addition of the fungus extract.

	Dark brown forest soil—surface			Dark brown forest soil—deep			Saline-alkali soil		
	Control	Addition of the fungus extract	Variation%	Control	Addition of the fungus extract	Variation%	Control	Addition of the fungus extract	Variation%
C : O	0.16	0.18	9.58	0.25	0.24	−3.95	0.31	0.23	−25.68
C : N	7.30	8.10	10.96	6.30	6.50	3.17	12.00	8.80	−26.67
C : Si	0.57	0.62	8.77	0.90	0.84	−6.67	1.03	0.77	−25.24
Si : Ca	13.60	13.60	0.00	17.90	20.50	14.53	6.10	7.10	16.39
C : Ca	7.70	8.40	9.09	16.20	17.10	5.56	6.30	5.50	−12.70
